# Enhancing cold and drought tolerance in cotton: a protective role of *SikCOR413PM1*

**DOI:** 10.1186/s12870-023-04572-6

**Published:** 2023-11-18

**Authors:** Mei Wang, Lepeng Wang, Xiangxue Yu, Jingyi Zhao, Zhijia Tian, Xiaohong Liu, Guoping Wang, Li Zhang, Xinyong Guo

**Affiliations:** 1 College of Life Science, Shihezi University, Shihezi, Xinjiang, 832000 People’s Republic of China; 2 Xinjiang Agricultural Development Group Crop Hospital Co. LTD, Tumushuke, Xinjiang, 844000 People’s Republic of China; 3 Agricultural Science Institute of the seventh division of Xinjiang Corps, Kuitun, Xinjiang, 833200 People’s Republic of China; 4 Department of Preventive Medicine, School of Medicine, Shihezi University, Shihezi, Xinjiang, 832000 People’s Republic of China

**Keywords:** Cold tolerance, Drought tolerance, Yield increase, Cotton, *SikCOR413PM1* gene

## Abstract

The present study explored the potential role of cold-regulated plasma membrane protein COR413PM1 isolated from *Saussurea involucrata* (*Matsum. & Koidz*)(*SikCOR413PM1*), in enhancing cotton (*Gossypium hirsutum*) tolerance to cold and drought stresses through transgenic methods. Under cold and drought stresses, the survival rate and the fresh and dry weights of the *SikCOR413PM1*-overexpressing lines were higher than those of the wild-type plants, and the degree of leaf withering was much lower. Besides, overexpressing *SikCOR413PM1* overexpression increased the relative water content, reduced malondialdehyde content and relative conductivity, and elevated proline and soluble sugar levels in cotton seedlings. These findings suggest that *SikCOR413PM1* minimizes cell membrane damage and boosts plant stability under challenging conditions. Additionally, overexpression of this gene upregulated antioxidant enzyme-related genes in cotton seedlings, resulting in enhanced antioxidant enzyme activity, lowered peroxide content, and reduced oxidative stress. *SikCOR413PM1* overexpression also modulated the expression of stress-related genes (*GhDREB1A*, *GhDREB1B*, *GhDREB1C*, *GhERF2*, *GhNAC3*, and *GhRD22*). In field trials, the transgenic cotton plants overexpressing *SikCOR413PM1* displayed high yields and increased environmental tolerance. Our study thus demonstrates the role of *SikCOR413PM1* in regulating stress-related genes, osmotic adjustment factors, and peroxide content while preserving cell membrane stability and improving cold and drought tolerance in cotton.

**Supplementary Information:**

The online version contains supplementary material available at 10.1186/s12870-023-04572-6.

## Background

Genomic breeding strategies can be used to design future crops. Improved varieties generated via such technologies will produce high yields with minimal agronomic inputs and will be better adapted to climate change [1]. During growth and development, plants are often challenged by abiotic stress factors, such as cold, drought, and salinity [2]. Among these, cold and drought are the most common and greatly impact cotton production and yields [3, 4]. Cold stress causes plant dwarfing, leaf decolorization, and reduced flowering [5]. It also affects pollen viability and pollen tube growth [6], reducing pollination and seed setting [7, 8]. On the other hand, drought stress causes stomatal closure, limits water transpiration [9], increases heat susceptibility, and minimizes nutrient absorption and photosynthesis [10] in plant, thereby slowing growth and reducing yield [11–13]. However, exposure to a small degree of abiotic stress in advance can improve the plant’s adaptability to subsequent stress [14].

Plants have evolved various regulatory mechanisms to cope with the changes in environmental conditions. Under normal conditions, plants produce a relatively low reactive oxygen species (ROS) and balance ROS scavenging and production [15]. However, stress can lead to an increase in ROS production [16]. Excessive ROS accumulation causes cell oxidative damage, inhibiting plant physiological processes [17, 18]. Superoxide (O_2_^−^) and hydrogen peroxide (H_2_O_2_) are the major ROS that act as signal transduction molecules mediating multiple stress tolerance [19]. Among these, H_2_O_2_ triggers the plant antioxidant system through abscisic acid (ABA), Melatonin (MET) and brassinosteroids (BRs), regulates cold stress, and improves cold tolerance [20–25]. Numerous studies have shown that the antioxidant defense mechanism of ROS scavenging is essential in resisting various stresses [26–28].

Plant responses to cold stress involve multiple levels of regulation, including metabolic changes, such as the accumulation of sugars and amino acids, and the CBF/DREB pathway [29, 30]. CBF proteins bind to the c-repeat (CRT) *cis*-elements and activate cold-responsive (*COR*) gene transcription to improve cold tolerance [21, 31–34]. Thus, the CBF transcription factor is a positive regulator of cold signals. Numerous studies have demonstrated that the expression of the COR gene in plants is positively correlated with cold resistance [35–38].

The COR413 family encodes plant-specific multi-transmembrane proteins [39]. Based on the predicted intracellular localization, the COR413 protein family is divided into two subclasses: chloroplast COR413-TM/IM (thylakoid membrane/intima) and plasma membrane COR413-PM [39]. Studies have demonstrated the role of *COR413* in improving plant stress tolerance. For example, *AtCOR413IM* of *Arabidopsis thaliana* stabilizes the chloroplast membrane under cold stress [40]. Besides, the overexpression of *COR413-TM1* in rice enhanced drought tolerance [41], and that of tomato chloroplast *COR413* (*SlCOR413IM1*) in tobacco improved drought tolerance [42]. Similarly, COR413*-PM*, located in the plasma membrane, is also known to regulate plant stress tolerance. Overexpression of *SikCOR413PM1* isolated from *Saussurea involucrata (Matsum. & Koidz)* in tobacco enhanced the tolerance of transgenic tobacco to cold and drought stress [43]. The overexpression of *PsCOR413PM2* in Arabidopsis increased osmotic stress and cold tolerance [44]. *LeCOR413PM2* overexpression in the tomato also improved cold tolerance [45].

Cotton is one of the most important agricultural products in the world and is mainly grown in arid regions, such as tropical and subtropical regions [46]. Cotton is grown in over 80 countries [47] and is an important cash crop in China. Xinjiang, the main production area of cotton in China, is rich in land resources; however, the distribution of water resources in this region is extremely uneven, and the temperature is relatively low in early spring during the growth of cotton seedlings [48]. Therefore, much cotton is subjected to drought and cold stress at the seedling stage. Cotton fiber yield and quality are often not guaranteed after experiencing abiotic stresses such as cold and drought [49, 50]. Besides, the simultaneous occurrence of two or more abiotic stresses causes a much greater yield loss than a single stress [51]. Therefore, there is an urgent need to develop cotton cultivars with multiple stress tolerance and meet the increasing demand of people. Studies have demonstrated the use of transgenic methods to improve cotton tolerance. For example, Basso et al. (2021) overexpressed the *CaHB12* transcription factor in cotton and increased its drought tolerance [52]. Overexpression of *GthCBF4* by Liu et al. (2021) significantly improved the cold stress tolerance of cotton [53]. Zhang et al. (2021) found that overexpressing *GhEXLB2* in cotton enhanced drought tolerance at the germination, seedling, and flowering stages [54]. Similarly, overexpressing *AmCBF1* [55] and *GhGLK1* [56] increased cold and drought tolerance. On the other hand, *GhORP_A02* gene silencing enhanced the drought tolerance of cotton [57]. However, there is still a need to develop tolerant cotton genotypes to overcome cold and drought stress and maintain their yield and fiber quality.

Previously, overexpression of the *SikCOR413PM1* gene isolated from *S. involucrata* (Matsum. & Koidz) [58] improved stress resistance in tobacco [43]. However, most conventional methods to develop the stress tolerance require laborious characterization of the progenies of multiple generations derived from time-consuming genetic crosses [59]. In such cases, phenotypic analysis helps investigate gene function. Therefore, the present study explored the role of *SikCOR413PM1* overexpression in cotton based on the Liu et al. (2017) approach [60]. The study showed stronger stress tolerance of *SikCOR413PM1-*overexpressing cotton plants than wild-type (WT) plants in greenhouse and field trials. These findings provide a foundation for further research on *SikCOR413PM1* and its application in developing new cotton varieties.

## Results

### Identification of transgenic cotton plants overexpressing *SikCOR431PM1*

**Fig. 1:**
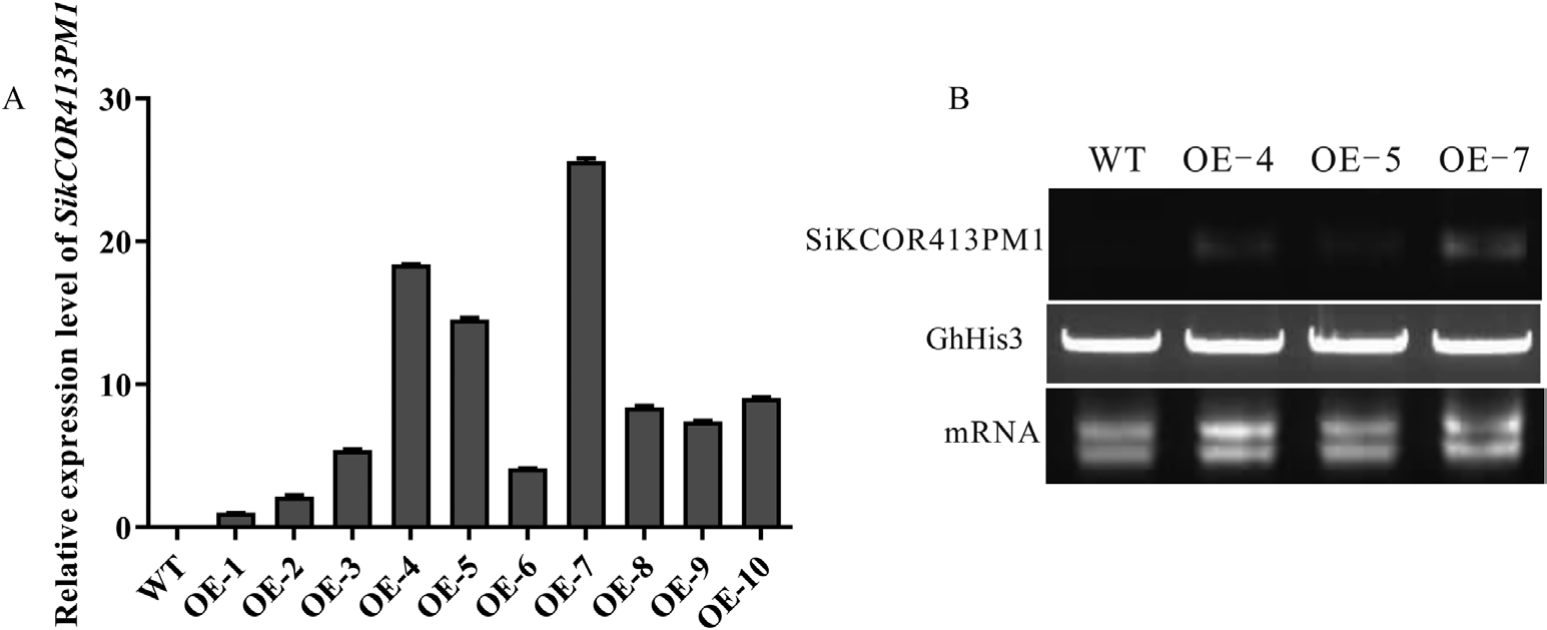
Expression levels of *SikCOR413PM1* in WT and transgenic cotton. **(A)** Quantitative real-time PCR and **(B)** semi-quantitative PCR show *SikCOR413PM1* expression in the WT and transgenic (OE-4, OE-5, and OE-7) cotton lines

To investigate the influence of *SikCOR431PM1* on cold and drought tolerance, we integrated this gene into cotton following the pollen tube channel method and generated transgenic plants. Ten transgenic cotton lines named OE-1 to 10 (We obtained ten transgenic cotton plants, which were defined as overexpression 1–10, or OE1-10 for short) were screened and analyzed by qRT-PCR (Fig. 1A). Among these lines, OE-4, OE-5, and OE-7 exhibited higher relative expression levels of *SikCOR431PM1* than the WT plants. Further analysis based on RT-PCR revealed expression levels consistent with qRT-PCR (Fig. 1B). Therefore, OE-4, OE-5, and OE-7 were selected for studying the gene function.The expression level at 0h (control time point) was defined as 1.0.

### Growth of *SikCOR413PM1-* overexpressing cotton seedlings under cold and drought stresses

**Fig. 2:**
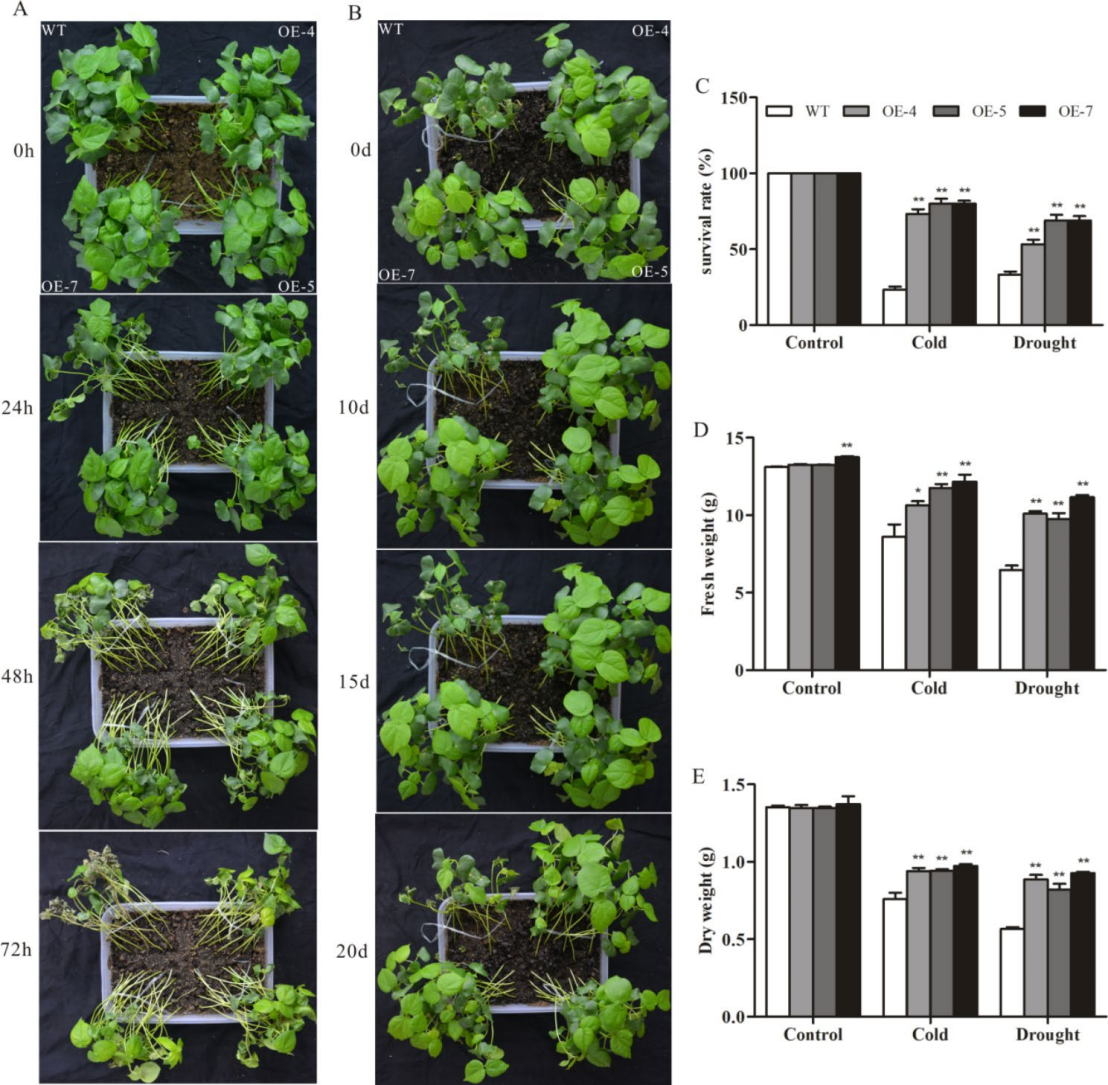
Phenotypic analysis of 20-day-old WT and *SikCOR413PM1-*overexpressing cotton seedlings under cold and drought stress. **(A)** Phenotype of WT and transgenic (OE-4, OE-5, and OE-7) lines under 4 °C for 24, 48, and 72 h; **(B)** Phenotype of WT and transgenic (OE-4, OE-5, and OE-7) lines under 20% PGE6000 stress for 10, 15, and 20 d; **(C)** Survival rate; **(D)** fresh weight and **(E)** dry weight of the plants under stress. The data shown are the average of three independent biological replicates. Bars represent standard deviations (SDs). * and ** indicate significant differences relative to the WT plants at P < 0.05 and P < 0.01, respectively. (In the cold and drought treatment figure (Fig. 2A&B), the upper left corner of the pot is the wild type, and the upper right, lower left and lower right corners are transgenic (OE-4, OE-5, and OE-7) lines, respectively)

The WT and transgenic cotton seedlings were exposed to cold (4 °C) and drought (20% PEG6000) stresses to investigate the role of *SikCOR413PM1*. The WT and transgenic lines grew well under normal conditions (25 °C). However, after exposure to 4 °C for 24 h, the leaves of WT cotton seedlings showed severe wilting, and most became necrotic and damaged. In contrast, the leaves of transgenic lines showed certain vitality and less wilting. After 48 h the leaves of wild-type plants withered much more than transgenic strain. After 72 h the whole wilted leaves of the wild-type plant turned brown, while the transgenic strain only showed yellowing and partial wilting of the leaves. (Fig. 2A). After treatment with 20% PEG6000 for 10days, the leaves of WT plants exhibited noticeable wilting and curling, while those of the transgenic lines showed less wilting. After 15 days, the WT lines exhibited more wilting and withering than the transgenic lines. After 20 days, a few WT plants exhibited even more severe growth retardation and leaf wilting [61, 62], whereas the transgenic lines showed less leaf wilting (Fig. 2B).

To further clarify whether *SikCOR413PM1* overexpression can improve the cytoprotection of cotton seedlings under cold and drought stress, we measured the survival rates of these plants (OE-4, OE-5, and OE-7) under cold and 20% PEG6000 stress. Under normal conditions, both WT and transgenic plants exhibited 100% survival. However, after exposure to 4 °C for 72 h, the survival rates of both WT and the transgenic lines decreased; the survival rate of WT decreased to 23.3%, while that of the transgenic lines OE-4, OE-5, and OE-7 decreased to 65.6%, 73.3%, and 80%, respectively. Similarly, after treatment with 20% PEG6000 for 20d, the survival rates of the WT and the OE-4, OE-5, and OE-7 transgenic lines were reduced to 33.3%, 61.1%, 53.3%, and 68.9%, respectively. These observations indicate a higher survival rate of *SikCOR413PM1*-overexpressing plants than the WT under cold and drought stresses (Fig. 2C).

Under normal conditions, no significant difference was observed in the fresh and dry weights between the WT and transgenic lines. However, after exposure to cold stress for 72 h, the fresh weight of OE-4, OE-5, and OE-7 was 23.6%, 36.6%, and 41.3% higher than that of the WT, respectively (Fig. 2D), while the dry weight was 23.7%, 24.1%, and 28.1% higher (Fig. 2E). Similarly, exposure to 20% PEG6000 stress for 20d resulted in a decline in the fresh and dry weights of both the WT and transgenic plants (OE-4, OE-5, and OE-7) compared with the untreated control. Nevertheless, OE-4, OE-5, and OE-7 lines exhibited 56.0%, 50.9%, and 72.5% higher fresh weight than the WT, and 56.5%, 44.7%, and 63.5% higher dry weight. These results indicate that *SikCOR413PM1* overexpression enhances the tolerance of cotton seedlings to cold and drought stress.

### Overexpression of *SikCOR413PM1* enhanced cold and drought tolerance of cotton seedlings

**Fig. 3:**
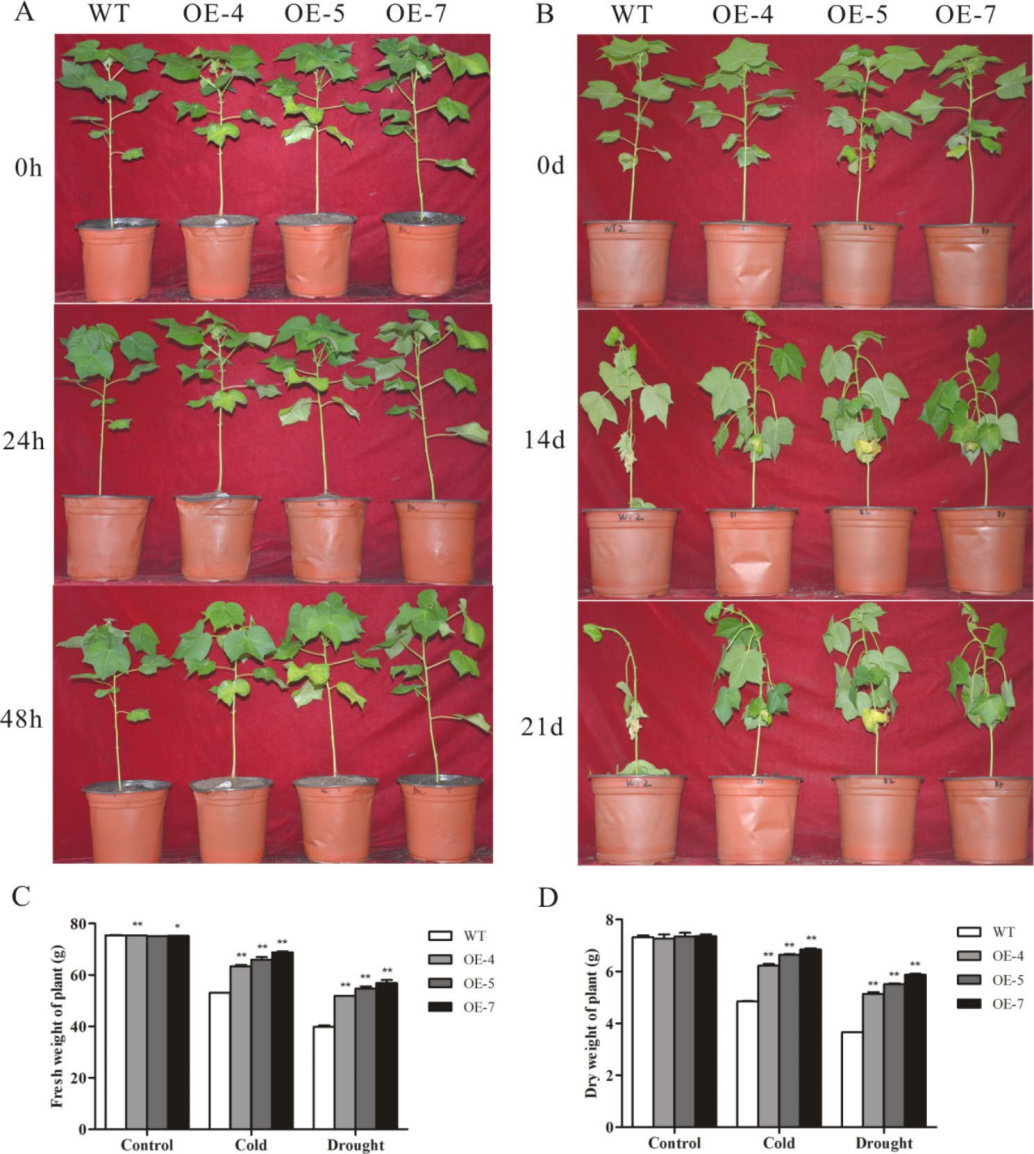
Phenotypic analysis of 70-day-old WT and *SikCOR413PM1*-overexpressing cotton Seedlings under cold and drought stress. **(A)** Phenotype of WT and OE-4, OE-5, and OE-7 transgenic plants after exposure to 4 °C for 24 h and 48 h; **(B)** Phenotype of WT and OE-4, OE-5, and OE-7 transgenic plants after exposure to drought for 14 and 21 days; **(C)** Fresh weight and **(D)** dry weight of the plants under stress. The data shown are the average of three independent biological replicates. Bars represent standard deviations (SDs). * and ** indicate significant differences relative to WT cotton plants at P < 0.05 and P < 0.01, respectively (In the phenotypic analysis figure of cotton cold stress, the cotton seedlings in each figure are wild type, OE-4, OE-5, and OE-7 transgenic plants from left to right)

The WT and transgenic cotton plants exhibited robust growth after 70 days at room temperature (25 °C) without treatment. However, after 24 h of exposure to 4 °C, the top leaves of the WT plants showed slight wilting, while those of the transgenics showed no significant change. After 48 h of exposure, the WT plants exhibited severe wilting of leaves at the top, while the transgenic plants showed only slight wilting and drooping of leaves (Fig. 3A). Similarly, after 14 days of exposure to drought, the WT plants showed apparent signs of wilting, with loose and yellow leaves near the base. In contrast, the leaves of transgenic cotton showed less wilting. After 21 days of exposure to drought, the WT plants exhibited severe growth retardation and leaf wilting and shedding. On the other hand, the trangenic plants’ leaves did not fall off; moreover, the transgenic stems remained full throughout the treatment period (Fig. 3B).

Under normal conditions, the WT and the transgenic plants showed no significant difference in average fresh and dry weights. However, after exposure to cold, the WT plants had 19.4%, 24.2%, and 25.5% lower fresh weight (Fig. 3C) and 28.3%, 36.9%, and 41.1% lower dry weight (Fig. 3D) than the transgenic lines OE-4, OE-5, and OE-7, respectively. Similarly, the WT and transgenic plants exposed to drought stress exhibited lower fresh weight and dry weight than the untreated control. Nevertheless, the OE-4, OE-5, and OE-7 transgenic lines had 30.1%, 37.2%, and 42.6% more fresh weight than the WT, and 40.6%, 50.6%, and 60.4% more dry weight. These findings suggest that *SikCOR413PM1* overexpression in cotton seedlings enhances the cold and drought tolerance.

### Overexpression of *SikCOR413PM1* alleviated the damage to the cell membrane and enhanced the accumulation of osmoregulatory substances

**Fig. 4:**
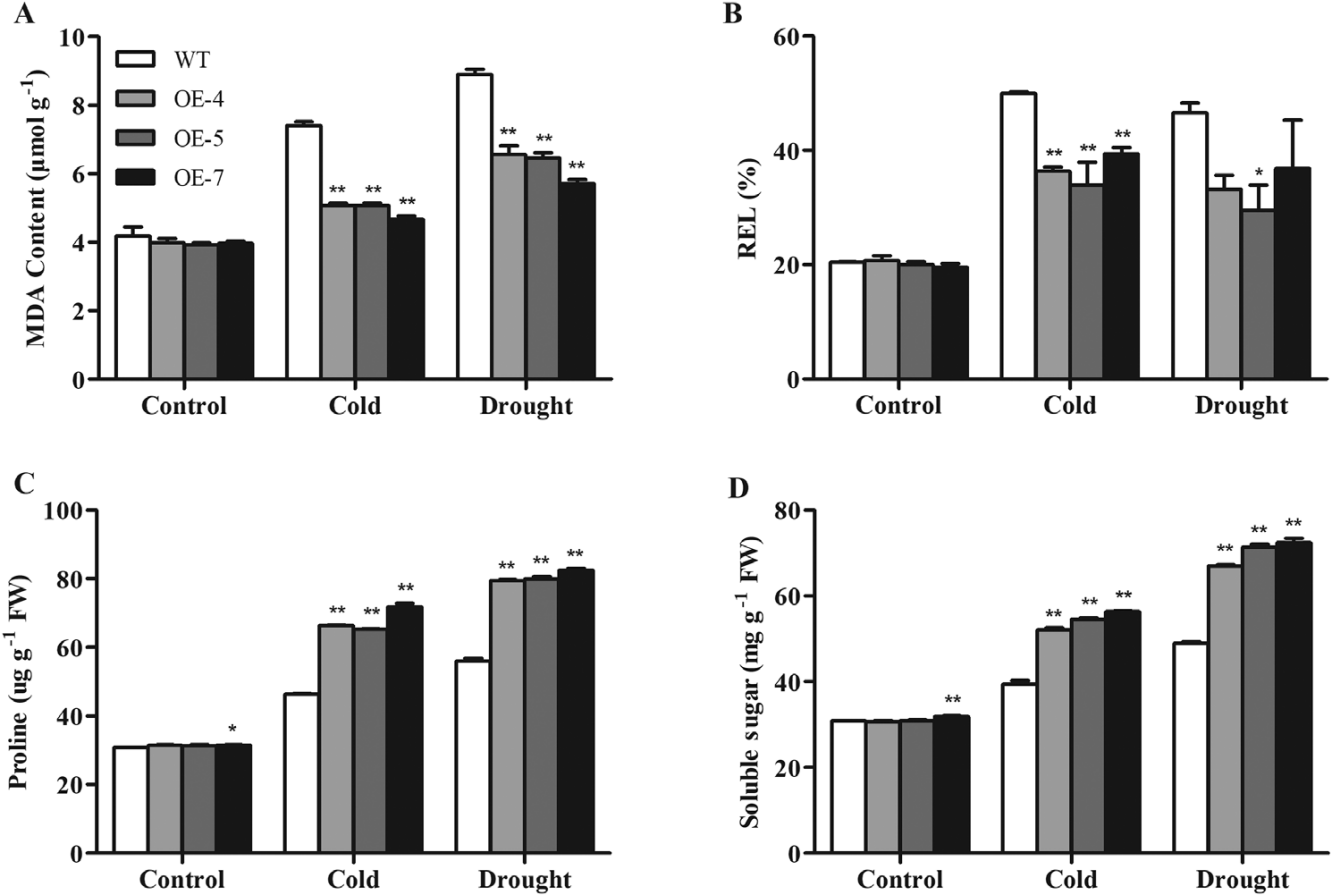
Physiological analysis of WT and *SikCOR413PM1*-overexpressing cotton plants after cold and drought stress treatments. **(A)** Malondialdehyde (MDA) content; **(B)** Relative electrolyte leakage (REL); **(C)** Proline content; **(D)** Soluble sugar content. The data shown for the WT are the means of three replicates, whereas those for the transgenic plants are means of three different lines. Bars represent standard deviations (SDs), * and ** indicate significant differences relative to WT cotton plants at P < 0.05 and P < 0.01

To further elucidate the possible physiological mechanisms through which *SikCOR413PM1* overexpression improves cell protection under cold and drought stress in cotton, we assessed the MDA content and relative conductivity of WT and transgenic plants (OE-4, OE-5, and OE-7). The MDA content in the leaves of transgenic plants was significantly lower than the WT plants under both stress conditions (Fig. 4A). Under 4 °C stress, the MDA content of OE-4, OE-5, and OE-7 was 31.7%, 31.6%, and 36.9% lower than that of the WT cotton. Under drought stress, the MDA content of OE-4, OE-5, and OE-7 was 26.3%, 27.5%, and 35.8% lower than that of WT cotton, respectively (Fig. 4B). Under normal conditions, the relative electrolyte leakage of both the WT and the transgenic lines was around 20%. However, the relative electrolyte leakage (REL) increased by 49.9%, 36.3%, 33.9%, and 39.4% after exposure to 4 °C and 46.6%, 33.2%, 29.5%, and 36.8% after drought treatment in the WT, OE-4, OE-5, and OE-7 plants, respectively.

Furthermore, the present study found low proline and soluble sugar levels in both WT and *SikCOR413PM1-*overexpressing plants before exposure to stress, and no significant difference between them (Fig. 4C and D). However, the levels of these two osmolytes significantly increased after exposure to cold and drought stress in WT and transgenic plants (OE-4, OE-5, and OE-7). Under 4 °C stress, OE-4, OE-5, and OE-7 exhibited 43.1%, 40.8%, and 55.1% higher proline levels and 32.0%, 38.6%, and 42.9% higher soluble sugar levels than the WT. Similarly, after drought treatment, OE-4, OE-5, and OE-7 had 41.8%, 42.7%, and 47.2% higher proline levels and 36.9%, 45.8%, and 47.9% higher soluble sugar levels than the WT. These observations indicate that *SikCOR413PM1* overexpression maintains the integrity of the cell membrane and promotes the accumulation of osmotic protective agents in cotton under stress.

### Overexpression of *SikCOR413PM1* maintained high antioxidant enzyme activity and gene expression

**Fig. 5:**
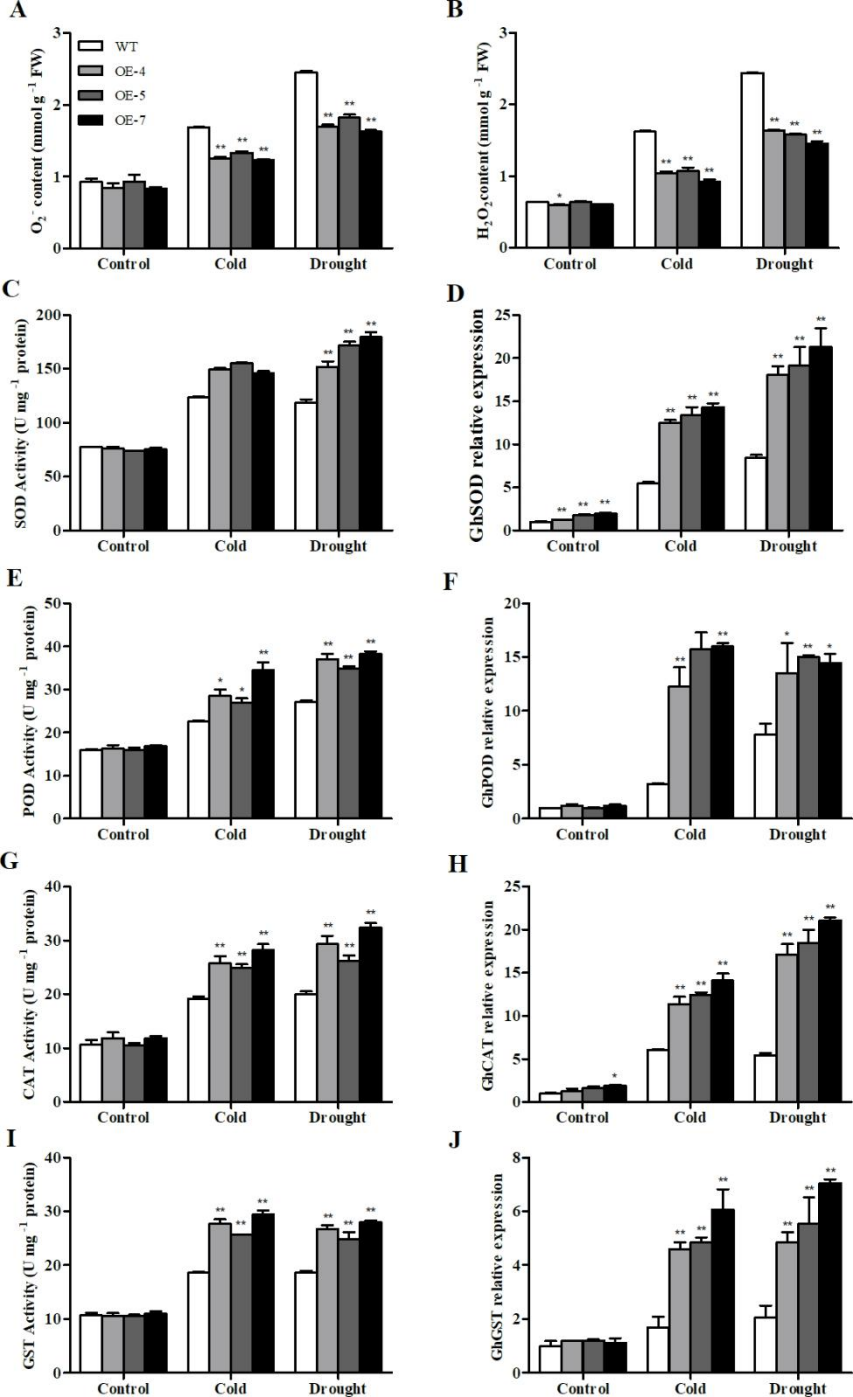
Reactive oxygen species (ROS), antioxidant enzyme activity, and antioxidant gene expression in WT and *SikCOR413PM1-*overexpressing cotton plants under cold and drought stress. **(A)** O_2_^−^ content; **(B)** H_2_O_2_ content; **(C)** SOD activity; **(D)** *GhSOD* expression; **(E)** POD activity; **(F)** *GhPOD* expression; **(G)** CAT activity; **(H)** *GhCAT* expression; **(I)** GST activity; **(J)** *GhGST* expression. The data shown for the WT are means of three replicates, whereas those for the transgenic plants are means of three different lines. Bars represent standard deviations (SDs). * and ** indicate significant differences relative to WT cotton plants at P < 0.05 and P < 0.01. The expression leve at 0h (control time point) was defined as 1.0

The study further analyzed the O^2−^ and H_2_O_2_ content in the WT and transgenic lines to assess their stress response. No significant difference was observed among the lines under normal conditions; however, after exposure to cold and drought stress, significant differences were detected between them. The O^2−^ and H_2_O_2_ levels in the transgenic lines were significantly reduced compared to those in the WT under stress (Fig. 5A and B).

We further measured the activity of the antioxidant enzymes superoxide dismutase (SOD), peroxidase (POD), catalase (CAT), and glutathione S-transferase (GST). Under normal conditions, no significant difference was observed in enzyme activity between the transgenic lines and the WT. Exposure to stress enhanced the antioxidant enzyme activity in both plants. The transgenic lines exhibited higher antioxidant enzyme activities than the WT lines. Consistently, the expression levels of *GhSOD*, *GhPOD*, *GhCAT*, and *GhGST* in the transgenic lines were significantly higher than those in the WT lines after stress treatment (Fig. 5C–J).These observations indicate that *SikCOR413PM1* overexpression increased the expression of genes encoding the antioxidant enzymes and enhanced the activity of the corresponding enzymes to regulate the amount of ROS and minimize the effect of oxidative stress.

### Analysis of stress-related gene expression in *SikCOR413PM1*-overexpressing cotton plants

**Fig. 6:**
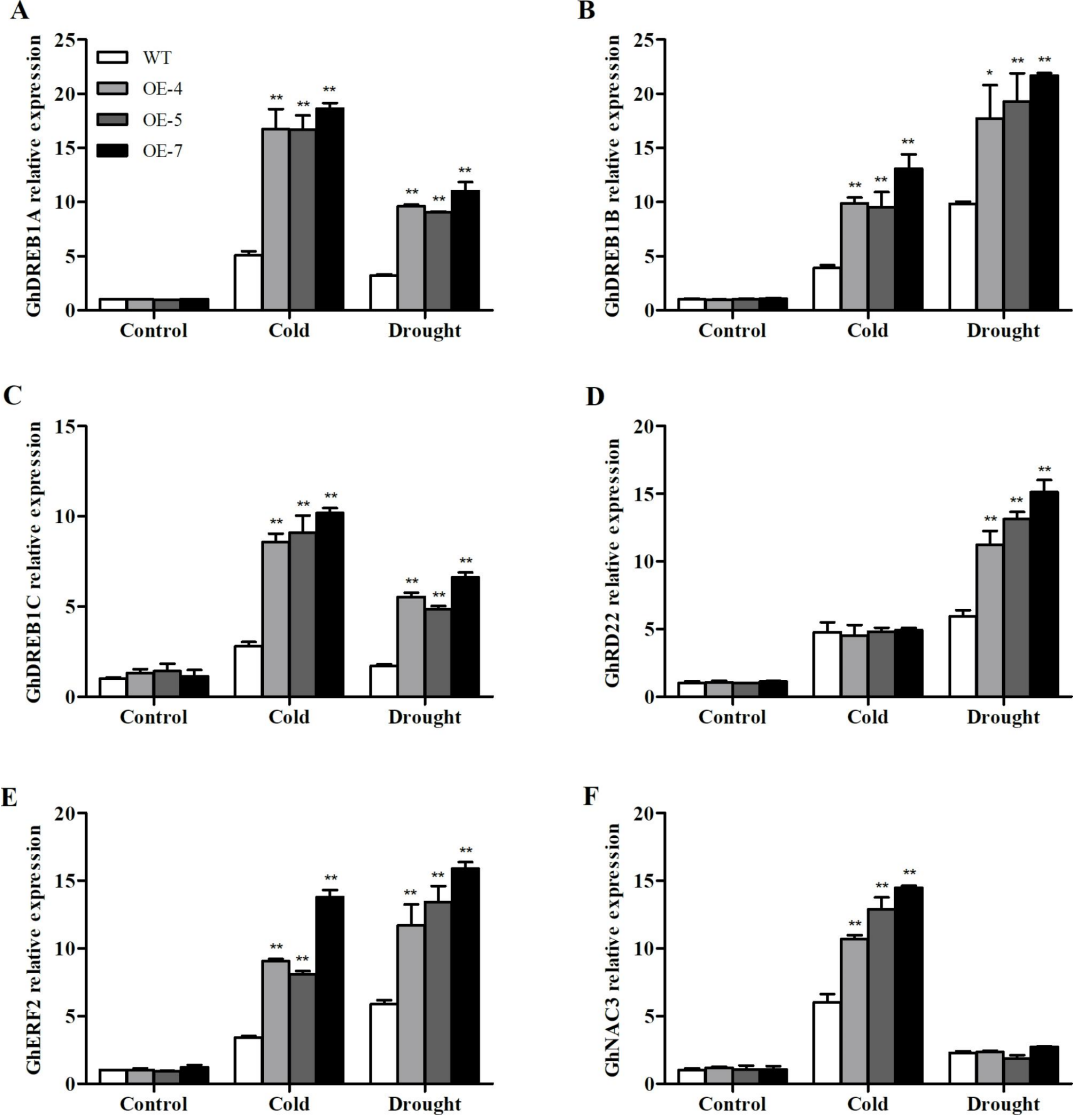
Relative expression of stress-related genes in WT and *SikCOR413PM1-*overexpressing cotton lines under cold and drought stress. Expression levels of **(A)** *GhDREB1A*; **(B)** *GhDREB1B*; **(C)** *GhDREB1C*; **(D)** *GhRD22*; **(E)** *GhERF2*; **(F)** *GhNAC3*, data shown are means ± SD of three replicates * and ** indicate significant differences relative to WT cotton plants at P < 0.05 and P < 0.01. The expression leve at 0h (control time point) was defined as 1.0

We subsequently analyzed the expression levels of several stress-related genes, such as *GhDREB1A*, *GhDREB1B*, *GhDREB1C*, *GhERF2*, *GhNAC3*, and *GhRD22*, in cotton plants. The analysis revealed no significant difference in the expression levels of *GhDREB1A*, *GhDREB1B*, and *GhDREB1C* between the WT and transgenic lines under normal conditions. However, after exposure to cold and drought stress, the expression levels of these genes in transgenic lines were significantly higher than those in the WT lines. Under cold stress, the expression levels of *GhDREB1A*, *GhDREB1B*, and *GhDREB1C* genes in transgenic lines were 16.7, 9.9, and 8.6 times (OE-4), 16.7, 9.5, and 9.1 times (OE-5), and 18.7, 13.1, and 10.2 times (OE-7) higher than those in the WT, respectively. The transgenic lines showed different expression levels of these genes after drought stress treatment. Compared with the wild type, the expression levels in OE-4 lines were increased by 9.6, 17.7, and 5.5-fold, respectively. The corresponding expression folds for OE-5 and OE-7 lines were 9.1, 19.3, 4.9, and 11.0, 21.7, and 6.6, respectively. (Fig. 6A–C). Under cold stress, no significant difference was observed in *GhRD22* expression between the transgenic and WT cotton. Under drought stress, the expression of *GhRD22* was significantly upregulated and was 11.2-fold (OE-4), 13.1-fold (OE-5), and 15.1-fold (OE-7) higher than that of the WT, respectively (Fig. 6D). Additionally, *GhERF2* expression in WT and transgenic plants was significantly different under stress treatment. The expression of *GhERF2* in transgenic plants was 9.1 and 11.7 times (OE-4), 8.1 and 13.4 times (OE-5), and 13.8 and 15.9 times (OE-7) higher than that in the WT under cold and drought stress, respectively (Fig. 6E). Under drought stress, no significant difference was observed in *GhNAC3* expression between transgenic and WT cotton. Meanwhile, after cold stress treatment, the expression levels increased in each plant. The expression levels of transgenic lines OE-4, OE-5, and OE-7 were 10.7, 12.9, and 14.5 times higher than those of the WT (Fig. 6F). Under cold stress, the transgenic lines exhibited significantly higher expression levels of all stress-related genes, except GhRD22. Similarly, under drought stress, the expression levels of all stress-related genes, except GhNAC3, were significantly higher in the transgenics than the WT. Besides, among the three transgenic lines, OE-7 exhbitied the highest expressionof these stress-related genes. These results indicate that *SikCOR413PM1* overexpression improves the expression of stress response genes and enhances the cold and drought tolerance of cotton.

### Agronomic traits of *SikCOR413PM1*-overexpressing cotton in the field

In the greenhouse, the plant height of transgenic cotton overexpressing *SikCOR413PM1* increased slightly but not significantly, and the number of fruit branches per plant and bolls per plant increased significantly compared with the WT (Table. S1). The *SikCOR413PM1*-overexpressing lines had eight to nine fruit branches per plant, while the WT had only seven. In addition, the transgenic lines had an average of one or two bolls per plant, while the WT had. Although no significant difference was observed in boll weight and lint weight between the transgenic lines and WT plants, the fiber yield of the transgenic lines was moderately higher than that of the WT. The seed cotton yield and lint yield of transgenic cotton were also higher than those of the WT. These differences probably resulted from the *SikCOR413PM1-*mediated multiple tolerance activities.

**Fig. 7:**
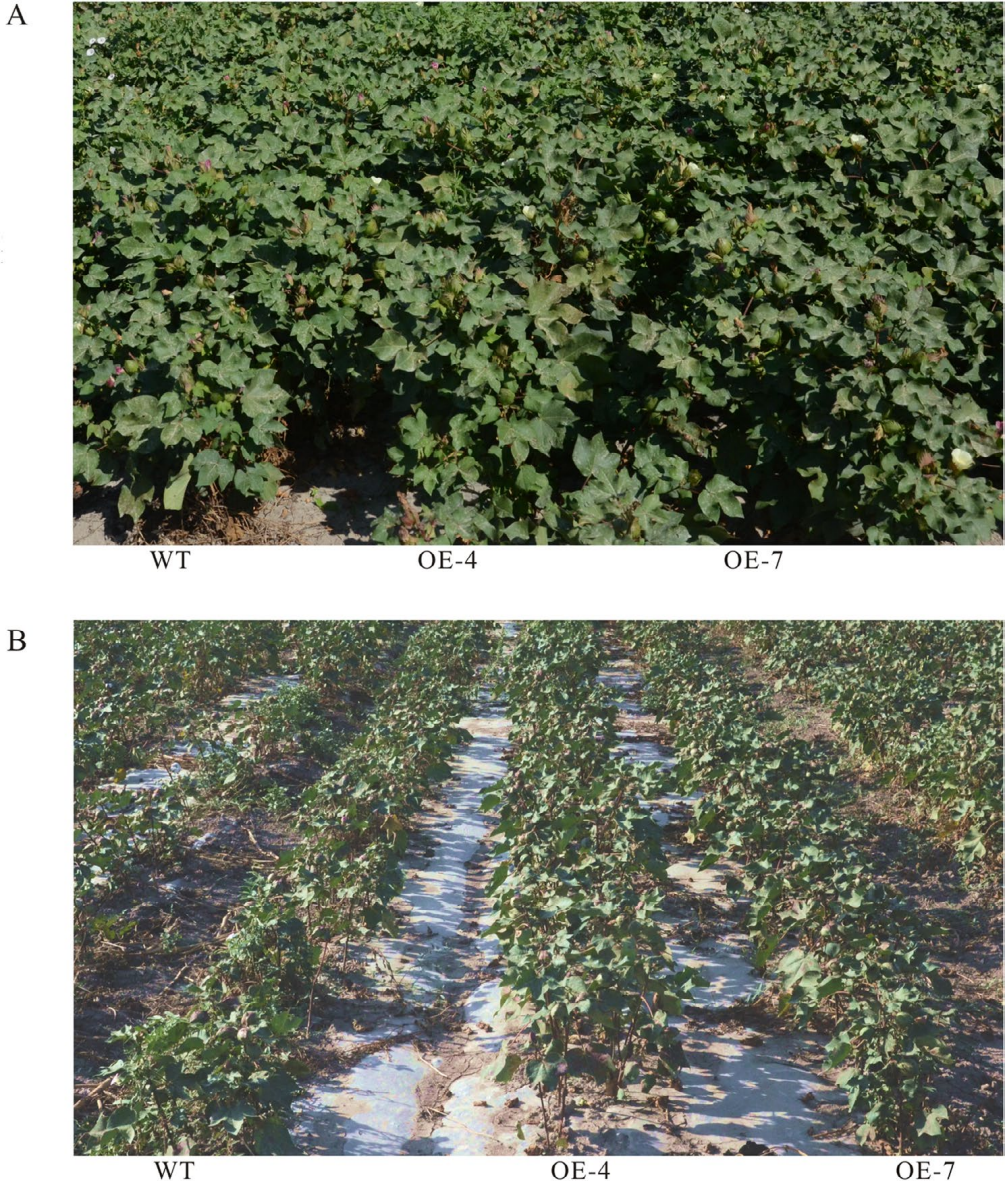
Phenotypic analysis of WT and *SikCOR413PM1-*overexpressing cotton lines under drought stress in the field. **(A)** Plants grown under normal conditions with regular irrigation. Photos were taken on 70 days after drought stress. **(B)** Plants grown under drought stress. Photos were taken on 70 days after drought stress

Further, we performed field trials to evaluate the drought tolerance of cotton plants overexpressing *SikCOR413PM1* (Fig. 7). The plant height, fruit branch number per plant, boll number per plant, and seed cotton yield of OE-4 and OE-7 lines were significantly higher than those of the WT. However, no significant difference was observed in the single boll weight, single boll lint weight, and lint percentage between the OE-4 and OE-7 lines. These results indicate that *SikCOR413PM1* overexpression could improve the tolerance and yield of cotton under drought stress in the field.

## Methods

### Plant materials and growth conditions

The Xinjiang upland cotton (*Gossypium hirsutum*) Xinluzao 17 was used in this study. The seeds of this cotton were provided by Li Baocheng, the vice president of the Xinjiang Academy of Agriculture and Reclamation Sciences. Under normal conditions, the WT and transgenic plants were maintained in a tissue culture laboratory under 60–70% relative humidity, 16 h/8 h light/dark cycle, and 25 °C average temperature. The seedlings were grown in plastic pots containing a composite matrix of nutrient soil, vermiculite, and perlite (1:1:1) and maintained in a culture chamber with a light intensity of 500 µmol^− 2^ s^− 1^ at a temperature range of 22–28 °C and relative humidity of 60–70%.

### Real-time quantitative PCR

Total RNA was isolated from the plant materials using the RNAiso Plus kit (TaKaRa) and treated with DNase I, following the manufacturer’s instructions. The first-strand cDNA was synthesized from the total RNA using oligo(dT) primers and PrimeScript® RTase (TaKaRa). Then, quantitative RT-PCR was performed on a LightCycler 480 instrument (Roche Diagnostics, Switzerland) with SYBR® Premix Ex TaqTM (TaKaRa, China) following the supplier’s instructions and MIQE guidelines to determine the expression patterns of *SikCOR413PM1* and various stress-related genes. Each reaction mixture (10 µL) contained 50 ng cDNA, 5 µL 2× SYBR® Green MasterMix reagent (Applied Biosystems), and 0.2 µM gene-specific primers (Table.S2). The qRT-PCR program involved pre-denaturation at 95 °C for 60 s, followed by 40 amplification cycles with denaturation at 95 °C for 15 s, annealing at 61 °C for 30 s, and an extension at 72 °C for 30 s, and pre-denaturation at 52 °C for 40 s. *GhHis3* (AF024716) was used as the reference gene, and the relative gene expression was calculated following the ΔΔCt method [63]. Each analysis was repeated three times.

### Transgenic cotton generation: a comprehensive overview of the process

Our previous work identified and sequenced the *SikCOR413PM1* gene. The WT plant without the exogenous gene were confirmed through molecular detection before the transformation, and the transgenic cotton plants containing *SikCOR413PM1* were obtained using the pollen tube pathway-mediated method in this study. The WT plants at the flowering stage were transformed, and the seed cotton from the bolls was harvested and labeled. These T0 seeds were planted in isolated and protected experimental fields during the second year, and the seedlings with expanded true leaves were screened for kanamycin (2 g/L) resistance. Further, DNA was extracted from the putative ones for detecting the transgene by PCR. From the positive transgenics, total RNA was extracted for qRT-PCR identification. The positive transgenic plants screened and identified were routinely managed and grown to blossom and produce bolls. Seeds were collected from these transgenics and sown on 1/2 MS medium containing kanamycin (80 µg/mL) for further screening. The resistant seedlings were transferred to soil for growth, and the seeds were collected at maturity for additional screening up to T4 generation.

### Plant stress treatments

Twenty-day-old WT and *SikCOR413PM1-*overexpressing transgenic cotton seedlings were subjected to 4 °C treatment for 24, 48, and 72 h and 20% PEG6000 for 10, 15, and 20 days. The phenotype of the cold-treated plants and the phenotype, the survival rate, fresh weight, and dry weight of the drought-treated plants were analyzed at regular intervals. Similarly, 70-day-old WT and *SikCOR413PM1*-overexpressing transgenic cotton plants were exposed to 4 °C for 24 and 48 h and 20% PEG6000 for 14 and 21 days. After both stress treatments, the plant phenotypic changes were recorded, and the leaves were collected from similar positions on the plants. And the collected samples were used for subsequent analysis of the fresh and dry weights, physiological indexes, enzyme activities, and gene expression levels.

### Investigation of agronomic traits

The plant height of selected WT and transgenic plants were measured using a tape, and the stem diameter using an electronic vernier caliper. The plant height was measured from the root neck to the apex. The stem diameter was measured 1 cm above the first leaf. The fresh weight and dry weight of the leaves. were measured using an electronic weighing scale.

### Analysis of the agronomic traits of cotton under drought stress in the field

Independent T0 transgenic plants were grown until maturity in the field. The inbred line seeds (T1) were harvested and sown in the experimental field. Further, based on the expression of the kanamycin resistance gene, T2 seeds were screened from the positive T1 lines. The homozygous transgenic lines were selected for selfing to obtain the T3 generation. This selection was carried out until the T4 generation to obtain a stable transgenic line. The performance of T4 homozygous transgenic and WT cotton lines was evaluated during the growing season in normal and drought-prone areas.

The traits of the WT and transgenic plants under normal environmental conditions were analyzed in the experimental field of the Agricultural Biotechnology Key Laboratory of Shihezi University in Shihezi City, Xinjiang. After one month, the plants were exposed to drought stress. All field trials were carried out using a randomized complete block design with three replicates. Each transgenic cotton was planted in a test plot of about 10.25 m^2^, with 240 plants per field, and repeated three timesice. A plant spacing of 12.5 cm and a row spacing of 30 cm were adopted. All recommended plant protection measures were carried out from sowing to harvest. Further, 50 plants were randomly selected from each replicate for analyzing the agronomic traits, such as plant height, fruit branch number per plant, boll number per plant, boll weight, lint weight per boll, seed cotton yield per plant, lint yield per plant, lint percentage, seed cotton yield, and lint yield per plot. The height of the main stem at the boll opening stage was represented as the plant height. The vegetative branches and the fruit branches were manually separated from the main stem and counted. Bolls were counted to determine the bolls per plant. Besides, all naturally opened bolls were collected from a plot and dried in an oven at 37 °C. Then, the weight of 100 randomly selected the boll weight, lint cotton yield, unginned cotton yield and lint percentage was measured.

### Statistical analysis

GraphPad Prism (version 7.0; GraphPad Software, LLC225 Franklin Street. Fl. 26 BOSTON, MA 02110 USA) and SPSS software (version 11.0; SPSS, Chicago, IL, USA) were used for statistical analysis. The data were expressed as the mean ± standard deviation of three independent experiments. The graphs were plotted using CorelDRAW X4 SP2 software (version 14.0; CorelDRAW X4 SP2, Canada). Dunnett’s multiple comparison test assessed the differences between WT and transgenic lines. The differences between the transgenic lines and WT plants were considered statistically significant at P < 0.05 and P < 0.01.

## Discussion

Abiotic stresses are known to cause water deficit in plants [32, 64, 65]. However, plants have evolved physiological mechanisms to adapt to water shortage, such as accumulating various osmotic regulators and antioxidants and producing ROS [32, 66]. Under cold and drought conditions, they maintain cell water by accumulating osmotic solutes, regulating osmotic potential, and adjusting osmotic pressure [67, 68]. They also initiate defense mechanisms by increasing the concentration of sugars, proline, amino acids, and other substances, reducing cell osmotic potential and increasing water absorption [69].

In this study, the soluble protein and proline levels in the *SikCOR413PM1-*overexpressing lines were significantly higher than those in the WT under cold and drought stress (Fig. 4C and D). Generally, the stronger the osmotic adjustment ability, the higher the tolerance and adaptability to osmotic stress. Thus, these observations suggest that *SikCOR413PM1* overexpression enhances the environmental adaptability of cotton by improving the osmotic adjustment ability through the accumulation of osmotic adjustment substances.

Typically, any change in environmental conditions affects cell membrane function [70–72]. Nevertheless, plants stabilize cell membranes under cold stress to prevent freezing-induced dehydration [73]. Some studies have shown that soluble sugars and proline stabilize proteins and overall cell structure, enhance cell membrane integrity, and improve frost resistance [74–78]. In cotton, the soluble sugars and proline protect cell membranes from oxidative damage under various abiotic stresses [79]. Proline, the most common osmotic solute, prevents water loss, reduces mechanical damage, and enhances stress tolerance [75, 80, 81]. In the present study, *SikCOR413PM1* overexpression in cotton enhanced the accumulation of soluble sugars and proline under cold and drought environments (Fig. 4C–D), which probably improved cell membrane stability.

The cell membrane is the primary target of adverse stress, and maintaining membrane integrity and stability under abiotic stress is critical for tolerance [82]. Under cold stress, the structure and function of plant cell membrane change, and membrane lipid peroxidation occurs, increasing cell membrane permeability, ROS, and relative conductivity [83]. Thus, the content of MDA reflects the degree of membrane lipid peroxidation and cell damage [84], and relative conductivity indicates plasma membrane damage caused by freezing [83].

There were no significant differences between WT and transgenic plants under normal conditions compared to REL and MDA. However, under 4 °C and drought stress, the contents of MDA and REL increased to different degrees, and the growth of WT was higher than that of transgenic plants. MDA and REL reduce the degree of plasma membrane damage by increasing their own content, thereby improving the stress resistance of cotton. The experimental results showed that MDA and REL increased more in WT than in transgenic plant, which indicated that transgenic plant was more tolerant to cold and drought stress than WT. Plants can also enhance freezing tolerance by improving antioxidant mechanisms [65, 85]. Thus, the lower content of MDA in *SikCOR413PM1*-overexpressing cotton than that of the WT indicates reduced membrane lipid peroxidation and enhanced stress resistance.

Several studies have investigated whether *COR* genes are induced in response to water shortage and cold stress. In Arabidopsis, water shortage induced most COR genes [86–88]. Similarly, cold and drought stresses induced *COR25* to protect *Brassica napus* cells from dehydration [35]. Therefore, we speculate that drought and cold stresses induce *SikCOR413PM1* in cotton, and the upregulation protects cell membranes and reduces cell damage by regulating osmotic regulators, MDA, and relative conductivity.

Currently, research on *COR413* family genes is limited. However, Zhang et al. found that the overexpression of *LeCOR413PM2* located in the plasma membrane enhanced the tolerance of transgenic tomatoes to cold stress by protecting the membrane from cold damage [45]. Similarly, the overexpression of *SikCOR413PM1*, located on the plasma membrane, improved tobacco’s drought and cold tolerance by protecting cell membrane stability [43]. It has been predicted that *COR413* family proteins located in the plasma membrane (*COR413-PM*) have a film-forming function. These earlier reports suggested that *COR413* family proteins in the plasma membrane play significant roles in maintaining cell membrane stability, requiring further investigation.

Under extreme temperatures and water deficit conditions, plants experience cell dehydration, photosynthetic damage, and membrane oxidation [32]. The abiotic stresses interfere with photosynthetic electron transport and produce ROS, severely damaging the chloroplasts [67, 89–91]. The organelles with high oxidative activity, such as mitochondria, chloroplasts, and peroxisomes, act as the primary sources of ROS and generate O_2_^−^ and H_2_O_2_, leading to oxidative damage of proteins, DNA, and lipids [16, 18, 90, 92]. Therefore, regulating ROS can protect cells from oxidative damage [93, 94].

ROS signaling is an adaptive regulatory mechanism of plants to stressful environments [67]. Plants have established an efficient ROS scavenging system, including antioxidant enzymes and antioxidants, which reduce ROS-induced damage [95]. Cell-protective enzymes mainly include SOD, CAT, APX, and GR. Overexpression of genes encoding these enzymes improves plant ability to scavenge ROS and their tolerance to abiotic stress [96–99].

The study also detected O_2_^−^ and H_2_O_2_ in WT and transgenic lines under cold and drought stress. The transgenics had lower O_2_^−^ and H_2_O_2_ content than the WTunder cold and drought stress (Fig. 5A-B). These observations indicate that *SikCOR413PM1* overexpression enhances the plant’s ability to scavenge ROS. Moreover, all stress treatments enhanced the activity of antioxidant enzymes in plants, particularly the transgenic lines. The expression levels of *GhSOD*, *GhPOD*, *GhCAT*, and *GhGST* in transgenic lines were also significantly higher than those in the WT lines after stress treatment (Fig. 5C–J). These results collectively suggest that the overexpression of *SikCOR413PM1* increased the expression level of genes corresponding to antioxidant enzymes in transgenic cotton, thereby enhancing the activity of enzymes that regulate ROS to reduce oxidative stress. Therefore, the study concludes that *SikCOR413PM1* maintains high antioxidant enzyme activity and gene expression, reducing oxidative stress in cotton.

Previous studies have related cold-induced changes in antioxidant enzyme activity in the leaves with frost resistance in several cereals [100–102]. Overexpression of the SOD-encoding gene in tobacco and the APX-encoding gene in tomato increased their tolerance to cold [103, 104]. The antioxidant defense system is closely related to plant frost resistance [90, 95, 105]. Cold acclimation induces ROS accumulation, activating the antioxidant defense system [106]. Mariem Ben Abdallah et al. indicate that in olive, drought stress pre-exposure can maintain homeostasis by increasing the activity of ROS scavenge enzymes (such as guaiacol peroxidase, SOD, and CAT) and reducing H_2_O_2_ and MDA levels [107].Chen et al. (2015) [108] found that silencing *GbMYb5* resulted in higher oxidative stress in cotton under drought conditions due to decreased POD, SOD, and CAT activities. These reports further confirm that overexpression of *SikCOR413PM1* can improve the cold and drought tolerance of cotton by reducing oxidative stress.

Proline protects cells from increased stress-related ROS levels [67]. In a previous study, *P5CS* transgenic wheat accumulated higher proline than WT plants and showed lesser membrane lipid peroxidation during drought [109]. This study indicated that proline plays a role in reducing ROS damage during drought. Specifically, cold acclimation induces rapid proline accumulation by changing the activity of proline metabolic pathway-related enzymes, thereby improving frost resistance [110]. In this study, transgenic cotton used proline as an osmotic regulator to improve cold and drought tolerance. The proline content was higher in the transgenic plants under drought and cold stresses than in the WT plants. Thus, we speculate that *SikCOR413PM1* overexpression increased proline accumulation, reducing the impact of ROS on cells.

Furthermore, the DREB family of transcription factors has been shown to play an important role in cold adaptation-induced frost tolerance. These transcription factors induce the expression of cold and dehydration-regulated genes in plants [111–113]. Studies have reported enhanced expression of *DREB* genes under cold and drought conditions [114]. For example, cold stress induced *DREB1A*, *DREB1B*, and *DREB1C* expression in Arabidopsis [113]. Thus, the increase in the expression of *DREB* partly explains the resistance of plants to stress.

In this study, the expression levels of *GhDREB1A*, *GhDREB1B*, and *GhDREB1C* were significantly higher in transgenic lines than in WT lines after cold and drought stress treatment (Fig. 6A–C). The expression levels of *GhRD22*, *GhERF2*, and *GhNAC3* also increased under both stresses (Fig. 6D–F). These results indicate that drought and cold strongly induced *GhDREB1A*, *GhDREB1B*, and *GhDREB1C*, and the expression of the five stress-related genes in the transgenic cotton plants were higher than in WT. These observations suggest that *SikCOR413PM1* overexpression helps increase the expression level of stress response genes. In other words, the increase in the expression of *DREB1* in the transgenics indicates a stronger tolerance than the WT under cold and drought conditions. In Arabidopsis, the DREB1 protein activates *COR* genes, such as *COR15a* [115], *COR6.6* [116], and *COR47* [117], and DREB1 protein plays a functional role in enhancing tolerance to cold and drought stress. Therefore, it can be speculated that stress increases the expression of *DREB1*, which may be related to the activation of the *COR413* gene. In summary, the overexpression of *SikCOR413PM1* promoted the expression level of stress genes, such as *DREB1*, thereby improving resistance to cold and drought stresses.

Overexpression of *SikCOR413PM1* positively influenced the growth of cotton under greenhouse conditions. Then, transgenic plants’ performance was studied in the field to investigate its actual impact on cotton production. The transgenic cotton had significantly higher seed and lint yields in the field, indicating its resistance to yield loss under stress. Although the growth status of the two lines did not differ significantly under normal conditions, the OE-4 and OE-7 transgenic lines exhibited substantially better growth under drought conditions than the WT (Fig. 7). Moreover, the number of fruit branches per plant, number of bolls per plant, and seed cotton yield were also significantly higher in the transgenic cotton than the WT (Table. S1). These results suggest that *SikCOR413PM1* overexpression improved drought tolerance and yield of cotton. The findings also provide a theoretical foundation for developing cotton varieties with improved environmental adaptability and yield.

**Fig. 8:**
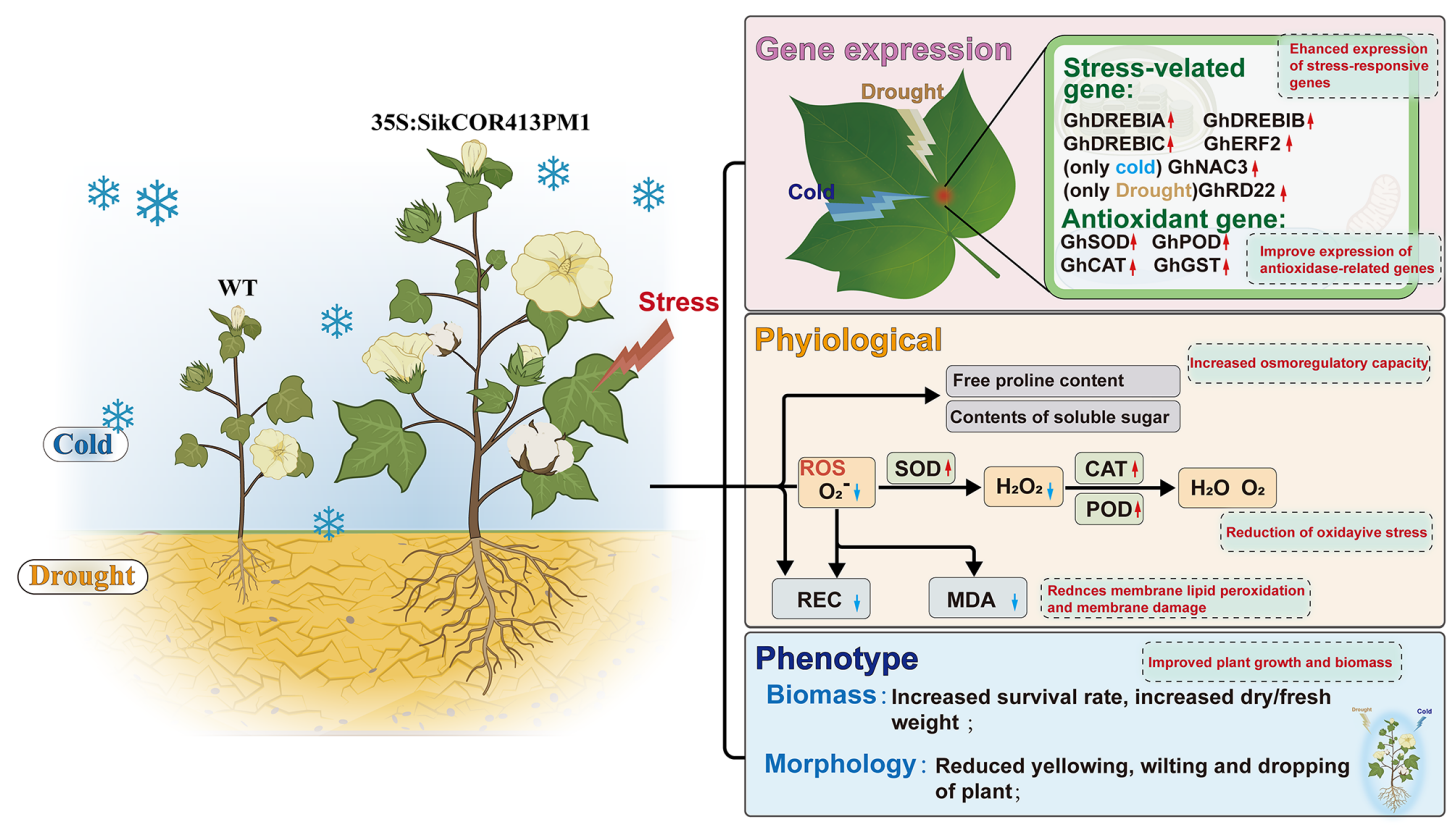
Illustration of the Mechanism by which the *SikCOR413PM1* Gene Mitigates Cold and Drought Stress

In nature, plants often face multiple abiotic stresses, significantly impacting growth and yield. So, to enhance cold and drought tolerance, the *SikCOR413PM1* gene was overexpressed in cotton plants. For stress analysis, three transgenic lines with high expression levels were selected (OE-4/OE-5/OE-7). The results demonstrated that overexpression of *SikCOR413PM1* improved cotton’s ability to tolerate cold and drought stress through various mechanisms. Overexpression of SikCOR413PM1 can accumulate osmotic substances by enhancing osmotic adjustment ability, improve ROS scavenging mechanism by increasing antioxidant enzyme activity and related gene expression, and maintain the integrity and stability of cell membrane by up-regulating stress-related genes, and finally alleviate the yellowing, withering, falling and waterlogging of leaves of transgenic lines in the face of low temperature and drought stress. Because the cell membrane of transgenic lines is more complete and stable, some plants can resume growth under normal conditions after stress. These findings indicate *SikCOR413PM1* is a candidate for breeding cotton varieties with improved cold and drought tolerance (Fig. 8). This research advances our knowledge and comprehensively explains how *SikCOR413PM1* enhances cotton’s resilience against cold and drought stresses. However, further studies are required to understand better the molecular mechanisms underlying *SikCOR413PM1*’s ability to mediate tolerance to cold and drought stress. These studies should focus on unraveling the signaling network and the specific functions of *SikCOR413PM1*.

The SikCOR413PM1 gene enhances cotton’s overall stress tolerance by upregulating stress-related genes and antioxidant enzymes. Furthermore, SikCOR413PM1 enhances cell osmotic regulation capabilities, while concurrently reducing cell membrane oxidative damage and membrane lipid peroxidation. This is achieved through increased antioxidant enzyme activity, elevated soluble substance levels, and reduced malondialdehyde content. Ultimately, these mechanisms lead to improved plant tolerance and increased crop yields.

### Electronic supplementary material

Below is the link to the electronic supplementary material.

Supplementary Material 1

## Data Availability

We submitted the gene sequence at NCBI, and since the gene is not publicly available, only the GenBank accession number of the nucleotide sequence provided by NCBI is available: BankIt2706231 Seq OR037196. Other GenBank accession numbers involved in the article are as follows: GhDREB1A:GenBank: AY321150.3. GhDREB1B: Ghi_A04G04116. GhDREB1C:GenBank: AYL99847.1. GhNAC3:GenBank: ACI15349.1. *GhRD22*:GeneID: 107,959,794. GhERF2:GenBank: DQ464375.1. GhHis3: GenBank: AF024716.

## Abbreviations

ABA: Abscisic acid
MET: Melatonin
BRs: Brassinosteroids
CAT: Catalase
CBF: C-receptor binding factor
COR: Cold-regulated
CRT: C-receptor
DREB: Dehydration response element binding factor
GST: Glutathione S-transferase
POD: Peroxidase
qRT-PCR: Quantitative Real-Time PCR
REL: Relative electrical conductivity
ROS: Reactive oxygen species
SOD: Superoxide Dismutase
MDA: Malondialdehyde
FT: Freezing tolerance
GR: Glutathione reductase
REL: Relative electrolyte leakage
