# Supplementary material for: Enhancing cold and drought tolerance in cotton: a protective role of SikCOR413PM1
Source: BMC Plant Biol. 2023 Nov 18;23:577. doi: 10.1186/s12870-023-04572-6 (PMC10656917; doi:10.1186/s12870-023-04572-6)
Supplement: Supplementary file 1 — Supplementary Material 1 [file 12870_2023_4572_MOESM1_ESM.docx]

Supplementary Material

**Enhancing cold and drought tolerance in cotton: A protective role of SikCOR413PM1**

Mei Wang^1^, Lepeng Wang^1^, Xiangxue Yu^1^, Jingyi Zhao^1^, Zhijia Tian^1^, Xiaohong Liu^3^, Guoping Wang^4^, Li Zhang^2^ & Xinyong Guo^1^*

^1^College of Life Science, Shihezi University, Shihezi 832000, Xinjiang, People’s Republic of China.

^2^Department of Preventive Medicine, School of Medicine, Shihezi University, Shihezi 832000, Xinjiang, People’s Republic of China.

^3^Xinjiang Agricultural Development Group Crop Hospital Co. LTD, Tumushuke 844000, Xinjiang, People’s Republic of China.

^4^Agricultural Science Institute of the seventh division of Xinjiang Corps, Kuitun 833200, Xinjiang, People’s Republic of China.

†Mei Wang and Lepeng Wang contributed equally as co-first authors.

*Corresponding author: Xinyong Guo & Li Zhang

College of Life Science, Shihezi University, Shihezi 832000, Xinjiang, People’s Republic of China.

Department of Preventive Medicine, School of Medicine, Shihezi University, Shihezi 832000, Xinjiang, People’s Republic of China.

* Correspondence: [guoxinyong2013@163.com](mailto:guoxinyong2013@163.com) & zl491191385@163.com

## Supplementary Figure


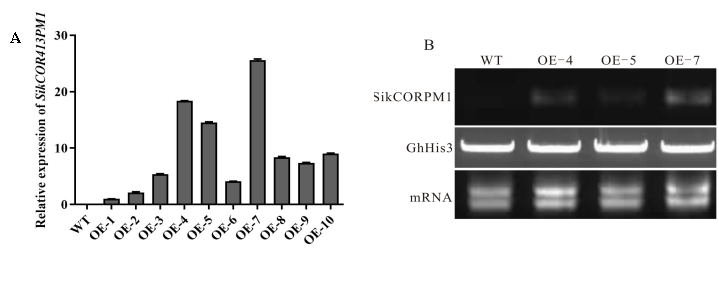


**Fig 1.Expression levels of *SikCOR413PM1* in WT and transgenic cotton lines.** (A) Quantitative real-time PCR analysis of relative expression of *SikCOR413PM1* in WT and ten transgenic lines; (B) Semi-quantitative PCR analyses of *SikCOR413PM1* expression in WT and three transgenic lines (OE-4, OE-5 and OE-7).

**WT OE-4 OE-5 OE-7 Marker MMMarker**


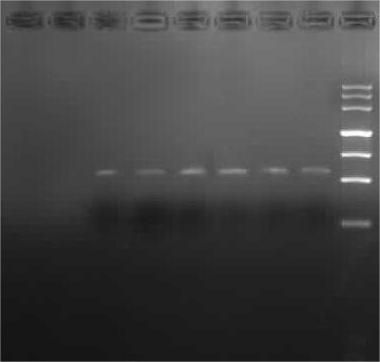


SikCOR413PM1

**Fig S1.****Semi-quantitative PCR analyses of** ***SikCOR413PM1* expression in WT and three transgenic lines (OE-4, OE-5 and OE-7).**

**Note:**Since the first gel electrophoresis pattern was too bright to accurately assess the levelof gene expression in different transgenic strains, we repeated the the electrophoresis by recovering the initial gel product. Consequently, we obtained a gel electrophoresis image displaying noticeable differences in gene expression, as depicted in the Supplementary Figure S1. This figure presents the results of semi-quantitative PCR analyses, specifically examining the expression of *SikCOR413PM1* in the WT, OE-4, OE-5, and OE-7 strains.

**WT OE-4 OE-5 OE-7**


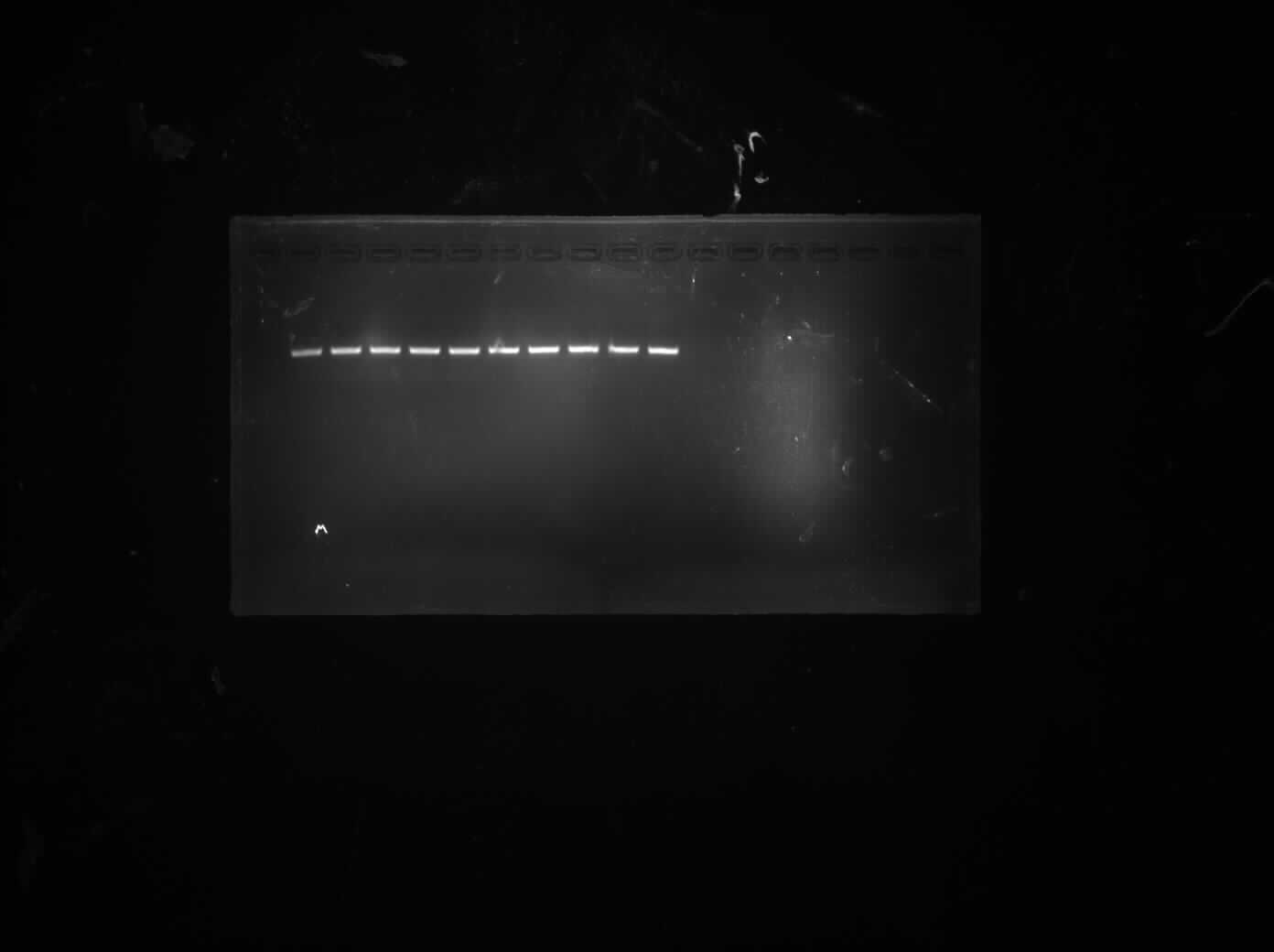


GhHis3

**Fig S2. Electrophoretic plot of the reference gene *GhHis3* in wild type and three transgenic lines (OE-4, OE-5 and OE-7).**

**Note:**Supplementary Figure 2 provides additional illustration of the gel electrophoresis pattern for the reference gene *GhHis3* as shown in Figure 1-B.*GhHis3* was used as the reference gene due to its relatively constant expression in the cells. This allowed for the correction of sample size and any errors in the sample loading process, ensuring the accuracy of the experimental results


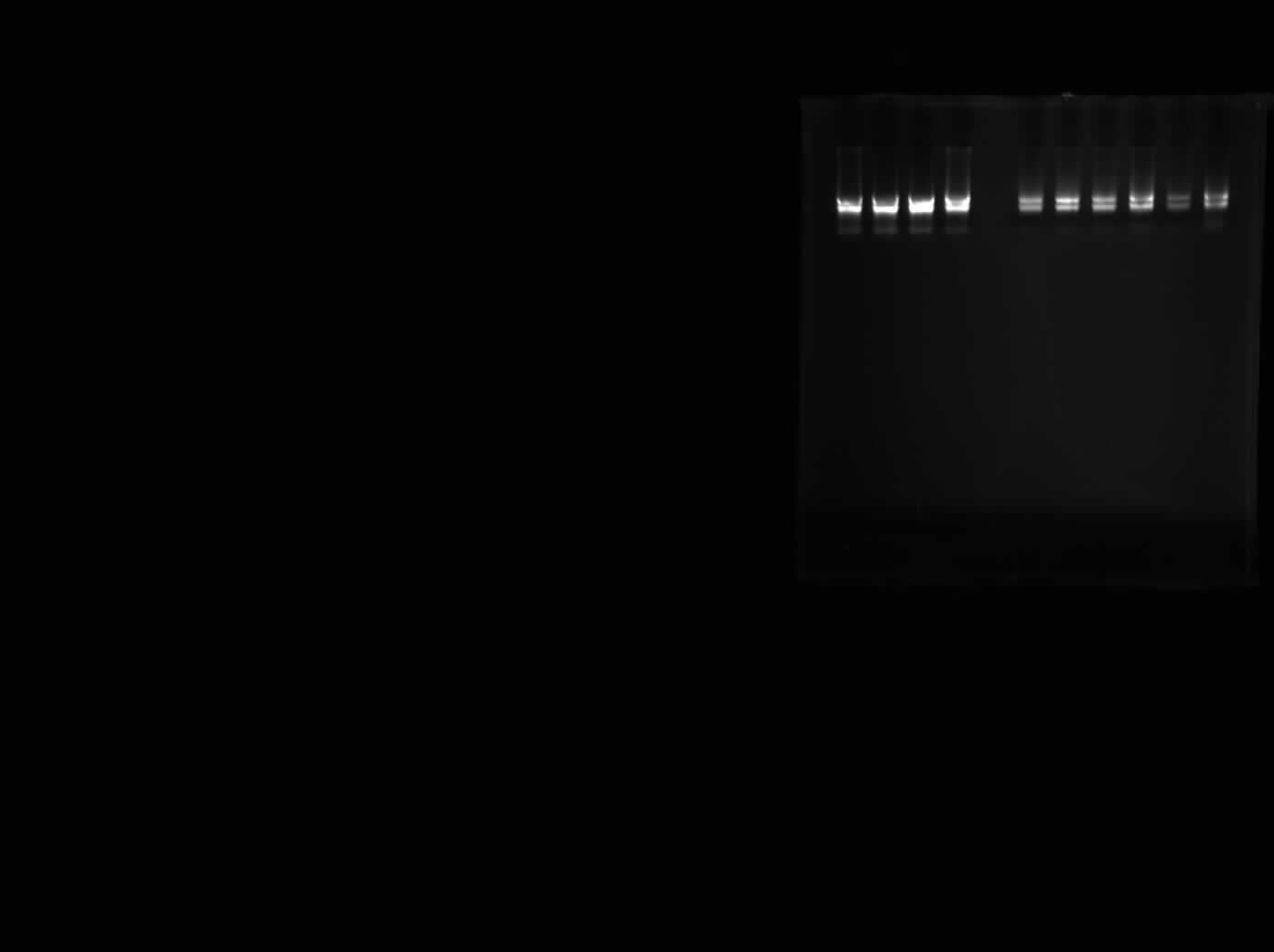


**WT OE-4 OE-5 OE-7**

mRNA

**Fig S3. Gel electrophoresis plot of *SikCOR413PM1* gene mRNA extraction.**

**Note:**Supplementary Figure 3 is a further illustration of the mRNA gel electrophoresis pattern in Figure 1- B. Gel electrophoresis picture of *SikCOR413PM1* gene mRNA extraction from wild type, OE-4, OE-5, and OE-7 different lines.

## Supplementary Table

**Table S1. Agronomic characteristics of WT and transgenic cotton lines under drought stress.**

| **Processing mode** | **Normal irrigation** | | | **Natural drought** | | |
| --- | --- | --- | --- | --- | --- | --- |
| **Genotype** | **WT** | **OE-4** | **OE-7** | **WT** | **OE-4** | **OE-7** |
| Plant height (cm) | 70.3±0.93 | 71.5±1.07 | 72.7±1.14 | 43±1.11 | 46.1±0.86* | 47.4±0.92** |
| Number of branches | 7.2±0.2 | 8.2±0.29** | 8.8±0.13** | 3.7±0.21 | 4.5±0.17* | 4.7±0.3** |
| Number of bolls | 7.1±0.31 | 8.4±0.16** | 9.1±0.18** | 2.7±0.15 | 3.7±0.21** | 4.2±0.25** |
| Boll weight (g) | 3.61±0.1 | 3.62±0.1 | 3.57±0.05 | 2.16±0.03 | 2.26±0.03 | 2.29±0.06 |
| Lint cotton yield (g/boll) | 1.38±0.07 | 1.36±0.02 | 1.37±0.01 | 0.65±0.01 | 0.67±0.02 | 0.71±0.01 |
| Unginned cotton  yield (g/plant) | 25.65±1.31 | 30.48±1.12** | 32.43±0.72** | 5.82±0.33 | 8.35±0.5* | 9.54±0.5** |
| Lint cotton yield (g/plant) | 9.95±0.99 | 11.44±0.28* | 12.44±0.23** | 1.75±0.11 | 2.48±0.15 | 2.97±0.17* |
| Lint percentage (%) | 38.54±2.47 | 37.86±1.29 | 38.45±0.69 | 30.05±0.71 | 29.9±1.12 | 31.06±0.59 |
| Unginned cotton  yield (Kg/plot) | 6.16±0.31 | 7.32±0.27** | 7.78±0.17** | 1.4±0.08 | 2±0.12* | 2.29±0.12** |
| Lint cotton yield (kg/plot) | 2.39±0.24 | 2.75±0.07* | 2.99±0.05** | 0.42±0.03 | 0.6±0.04 | 0.71±0.04* |

**Table S2. List of primers used in this study.**

| **Primer name** | **Primer sequence (5′-3′)** | **Purpose** |
| --- | --- | --- |
| *SikCOR413PM1* (*BamH* Ⅰ)-F | GGATCCATGATGAAGGGAGTAAAGAAC | Cloning |
| *SikCOR413PM1* (*Sal*Ⅰ)-R | GTCGACCTAAAATATTAAGCCGATCAAAGC | Cloning |
| *SikCOR413PM1*-qF | TGTGGTGGAGCAGATCAAGG | qPCR |
| *SikCOR413PM1*-qR | TATGTCCATCGGCGACACCATGA | qPCR |
| *SikGAPDH-qF* | TAGCAAGGATGCTCCCATGTT | qPCR |
| *SikGAPDH-qR* | GGAGCAAGGCAGTTGGTTGTG | qPCR |
| *GhDREB1A*-qF | GGATTTGGCTTGGAACTTTCCCGAA | qPCR |
| *GhDREB1A*-qR | CATCGTTTCTAGAATTCCCGCTCGA | qPCR |
| *GhDREB1B*-qF | CCGACCCAAAGGATATCCAAAAGAC | qPCR |
| *GhDREB1B*-qR | CGCTTCTTCGTCCAAGTAAAACCCTT | qPCR |
| *GhDREB1C*-qF | CTCAGATCCTTTTAGTGCGGAGTTG | qPCR |
| *GhDREB1C*-qR | GATAGTTACAGAACCCTTGTTCTGAG | qPCR |
| *GhERF2*-qF | GAGGAAGAACCAGTATCGTGGAATC | qPCR |
| *GhERF2*-qR | GCGGAGTCTCGTTAGGGAAG TTCA | qPCR |
| *GhRD22*-qF | AGGAGGTGGTGGTGTAAACGTCAA | qPCR |
| *GhRD22*-qR | ATGAAACACGGATCTCCTCCCGAA | qPCR |
| *GhNAC3*-qF | GTAAATGTGCGTCTCAGTCTAT | qPCR |
| *GhNAC3*-qR | GGTCCACGTCGGCTAATC | qPCR |
| *GhSOD*-qF | ATGATGGTCCTACAACTGTGAATGT | qPCR |
| *GhSOD*-qR | GCGTACTTCA TCCTCAGGAGCA | qPCR |
| *GhPOD*-qF | CACATTGTTGCTCTTTCTGGTG | qPCR |
| *GhPOD*-qR | GGATCTGACAGGAGAACTTTGT | qPCR |
| *GhCAT*-qF | TATCTGCAACTCCCAGCCAAT | qPCR |
| *GhCAT*-qR | TTCTCTCGCC TTCCAGTGCA A | qPCR |
| *GhGST*-qF | GTTGCTAGCTCTGTTGGGTATTTC | qPCR |
| *GhGST*-qR | AGGGAAGTAGTTTGTCTCTTGGAG | qPCR |
| *GhHis3*-qR | TGGGAAGGCTCCAAGGAAGCA | qPCR |
| *GhHis3*-qF | CGAGCCAACTGGATGTCCTTG | qPCR |

The primer sequences for stress-related genes and reference genes are referenced from the literature of Zhang et al. and HAO et al [1,2].

**Reference**

1. HAO, Yu-qiong, et al. Overexpression of AmDUF1517 enhanced tolerance to salinity, drought, and cold stress in transgenic cotton. Journal of Integrative Agriculture 17.10 (2018): 2204-2214.
2. Zhang L, Tian W, Huang G, Liu B, Wang A, Zhu J, Guo X. The SikCuZnSOD3 gene improves abiotic stress resistance in transgenic cotton. Mol Breed. 2021 Mar 10;41(3):26.
